# Supplementary material for: Persistent representation of a prior schema in the orbitofrontal cortex facilitates learning of a conflicting schema
Source: Nat Commun. 2026 Feb 10;17:2610. doi: 10.1038/s41467-026-69330-2 (PMC13003079; doi:10.1038/s41467-026-69330-2)
Supplement: Supplementary file 1 — Supplementary Information [file 41467_2026_69330_MOESM1_ESM.pdf]

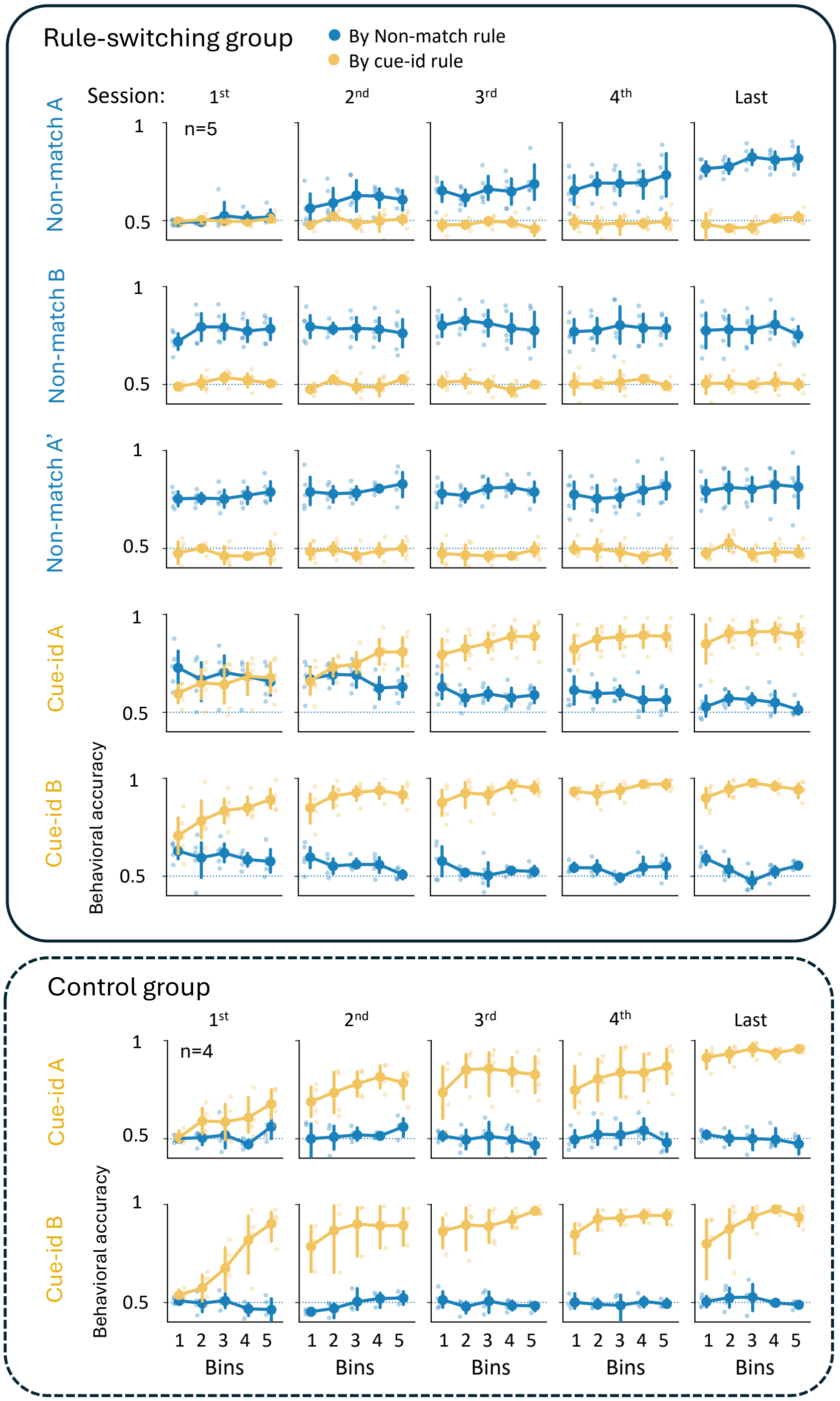

Supplementary Figure 1. Within-session accuracy dynamics by rule type. *Accuracy according to the non-match (blue) and cue-identity (yellow) rules broken down to different time bins within a session (data are shown as mean ± SEM with individual data points overlaid).*

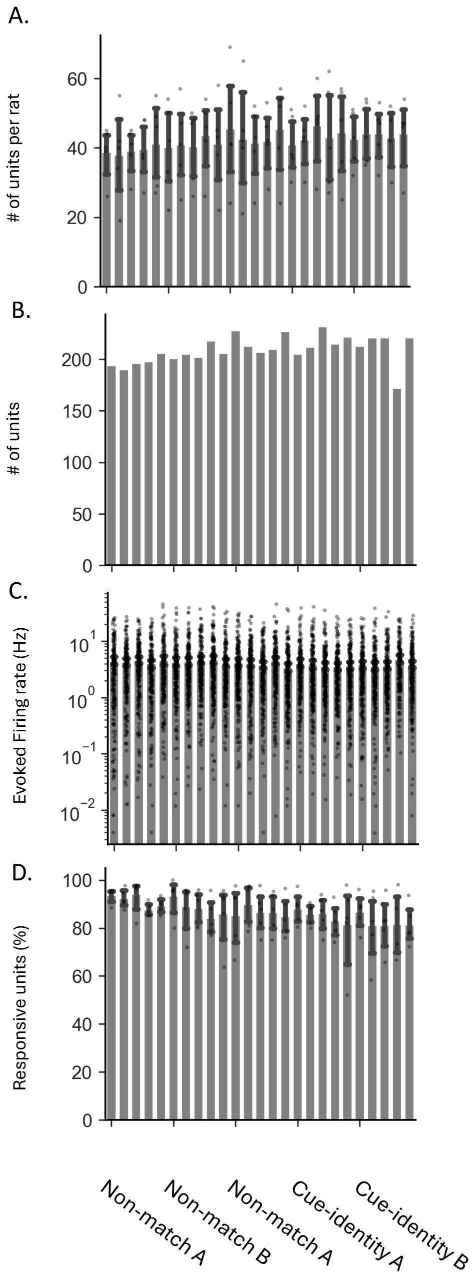

Supplementary Figure 2. Neural recording stability across sessions. A. *Single units recorded per rat per session (mean ± SEM). One-way ANOVA showed no significant session effect (F(24, 99) = 0.187, p = 1.000).* ***B.*** *Total single units per session were stable across sessions; no monotonic trend was detected (Spearman ρ = −0.217, p = 0.29; Kendall τ = −0.141, p = 0.3258).* ***C.*** *Firing rate in the 500 ms preceding the decision (mean ± SEM). One-way ANOVA did not detect a significant session effect (F(24, 5210) = 1.518, p = 0.0503).* ***D.*** *Percentage of units with a significant response during the odor-sampling period (mean ± SEM). One-way ANOVA indicated no significant session effect (F(24, 99) = 0.919, p = 0.576).*

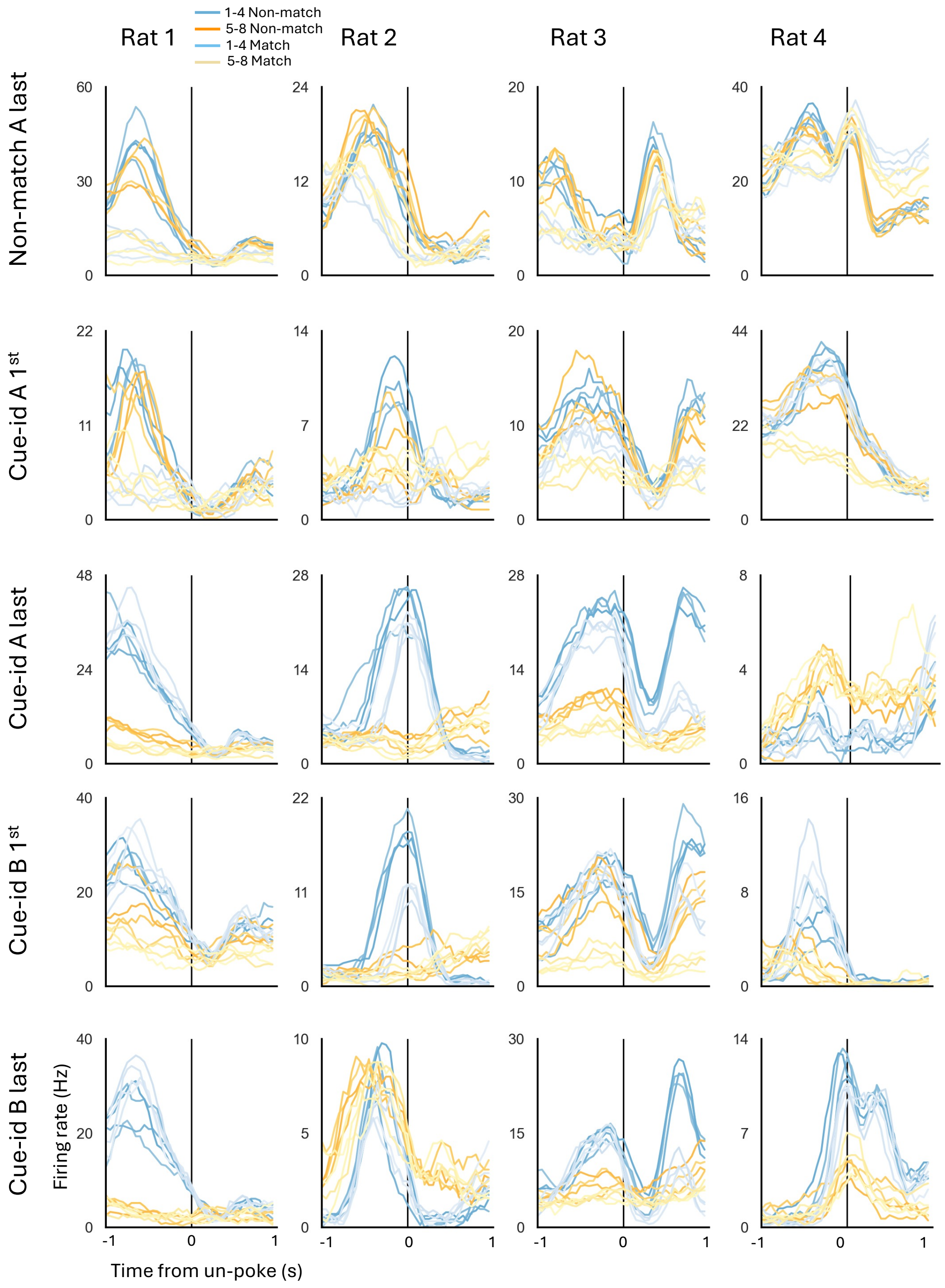

Supplementary Figure 3. Example neuronal PSTHs across odor types and trial configurations. *Peristimulus time histograms (PSTHs) of example neurons, from 4 rats and 5 learning sessions, in response to 16 different trial types: 8 odors (1-4: blue lines; 5-8: yellow lines) X 2 trial configurations (match: light colors; non-match: dark colors. The PSTHs were aligned to the decision time (un-poke from odor port).*

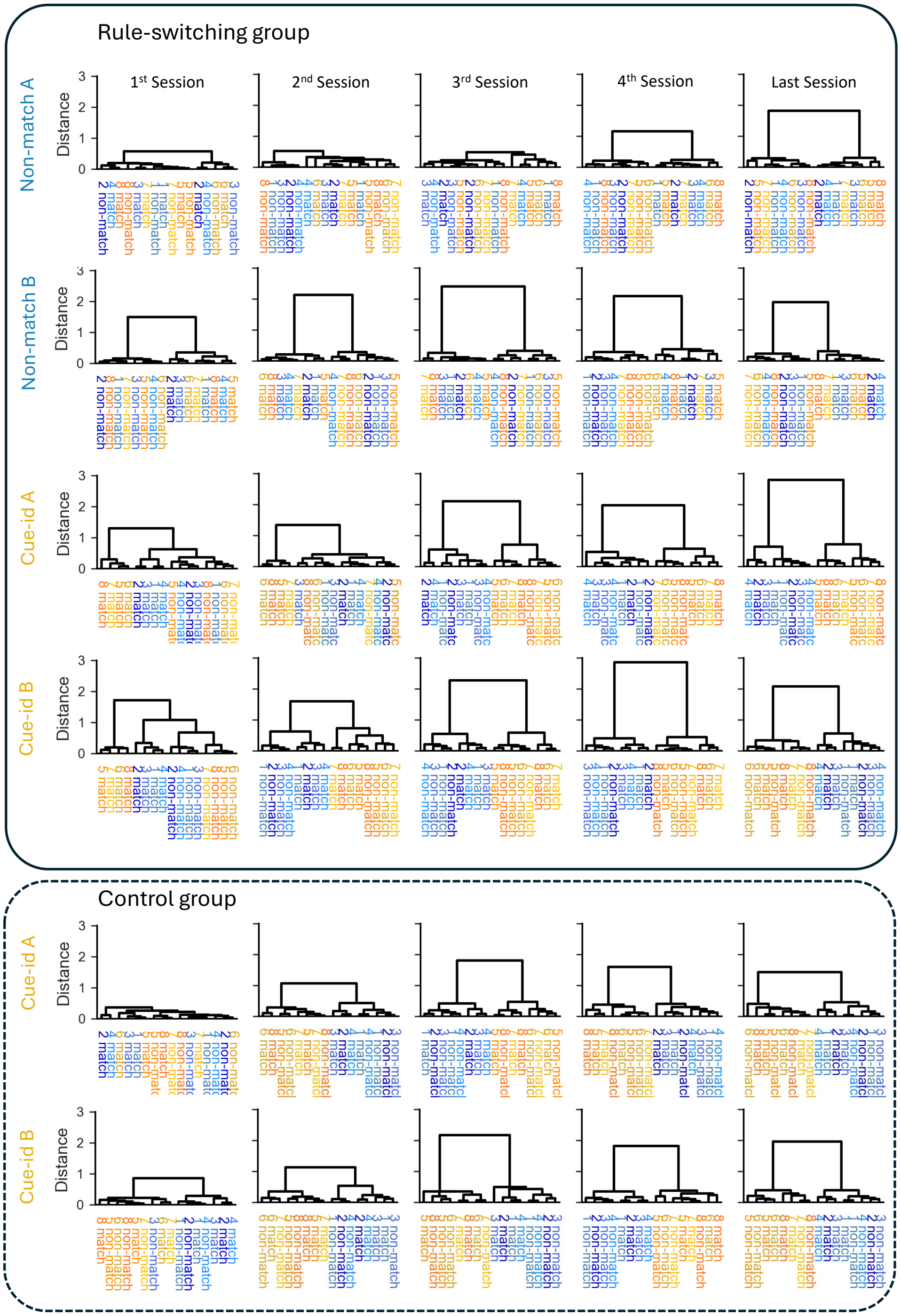

Supplementary Figure 4. Session-by-session dendrograms of trial-type clustering. *Session-by-session hierarchical dendrograms showing clustering of trial types across training, constructed from pairwise distances in UMAP space.*

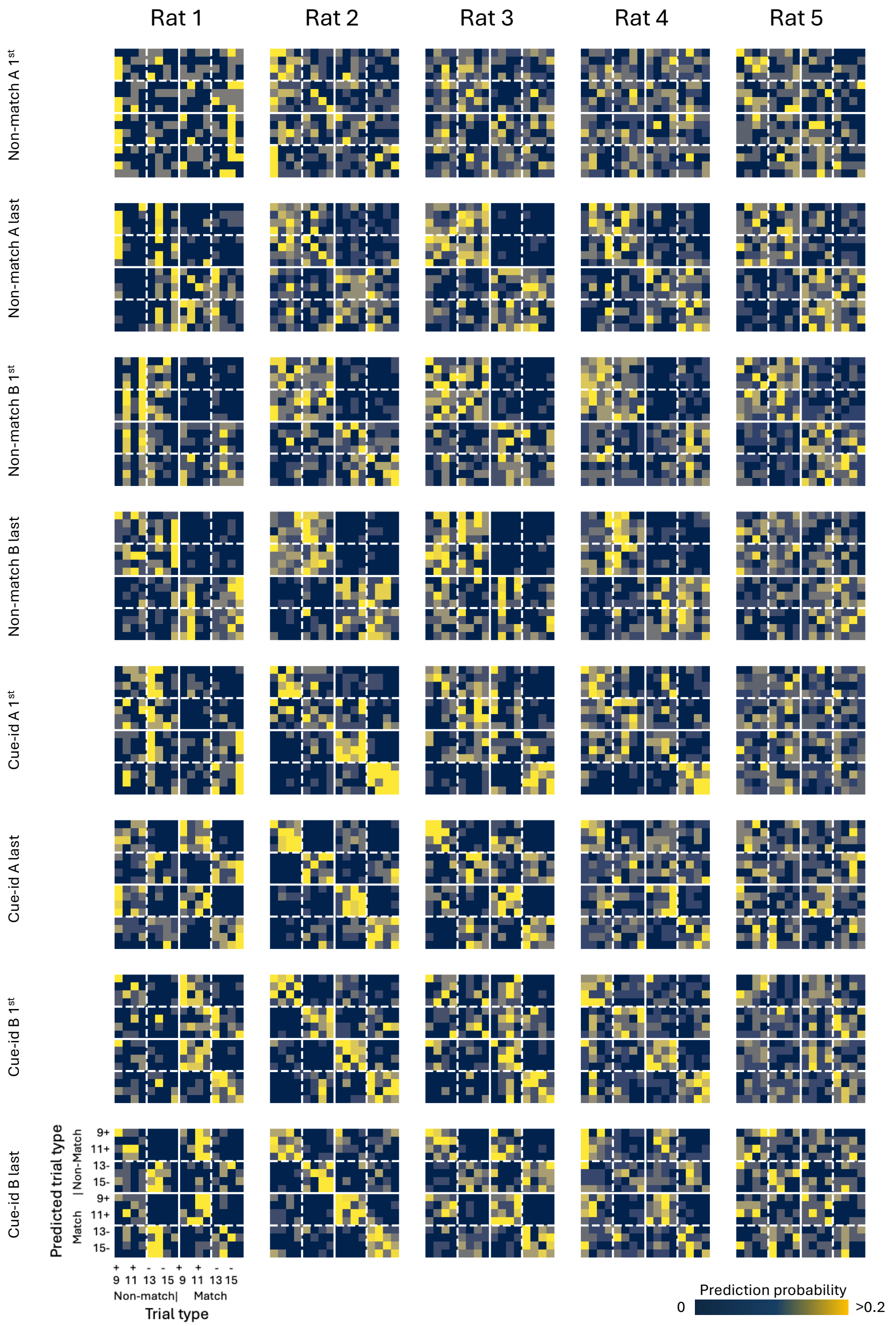

Supplementary Figure 5. Neural classifier confusion matrices across rats and training sessions. *Confusion matrices showing classifier performance for each rat (columns) across different training sessions (rows). Each matrix is based on a support vector machine (SVM) decoder trained on the neural population activity of that rat in a given session. Within each matrix, rows correspond to predicted trial types and columns correspond to true trial types, with values indicating classification probabilities.*

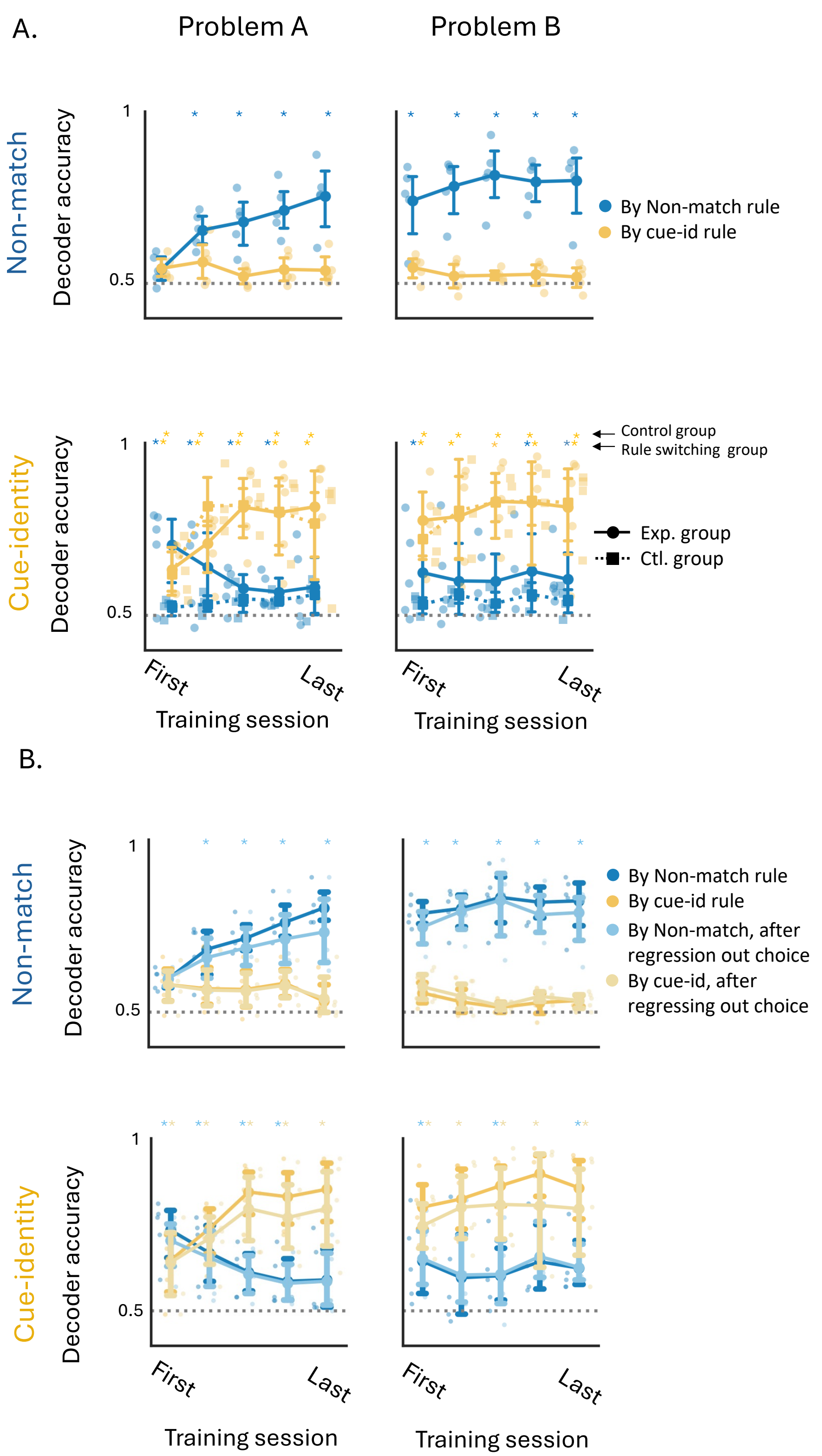
A.
Problem A
Problem B
Non-match
Decoder accuracy
1
0.5
By Non-match rule
By cue-id rule
Cue-identity
Control group
Rule switching group
Exp. group
Ctl. group
First
Last
Training session
B.
By Non-match rule
By cue-id rule
By Non-match, after regression out choice
By cue-id, after regressing out choice

Supplementary Figure 6. Neural decoder accuracy for rule representations. A. *Neural decoder accuracy based on the non-match rule (blue) and the cue-identity rule (yellow) in the main group of rats (circles, solid lines) and the control group (squares, dashed lines). Classifiers were trained separately for each rat and each session, and decoder accuracy was assessed using leave-one-out cross-validation within session. Data are shown as mean ± SEM with individual data points overlaid. Asterisks indicate statistically significant differences from shuffled data for the non-match rule (blue) and the cue-identity rule (yellow), based on combined p-values from 1000 permutation tests using Stouffer's Z-score method and corrected for multiple comparisons (*$p < 0.001$, asterisks in the upper row indicate statistically significant differences in the control group; see Supplementary Table 15).* ***B.*** *Decoder accuracy based on the non-match rule (blue) and cue-identity rule (yellow), shown before (dark lines) and after (light lines) regressing out the rats' choice (go/no-go). Asterisks indicate statistically significant differences of the data following regression, from shuffled data for the non-match rule (light blue) and the cue-identity rule (light yellow), based on combined p-values from 1000 permutation tests using Stouffer's Z-score method and corrected for multiple comparisons.*

Panel	Measure	Effect	df	F	p-value	Partial η^2	95% CI for η^2
D	Accuracy (Non-match)	Session	9, 40	10.95	2.3×10^{-8}	0.71	[0.82, 0.95]
	Accuracy (Cue-ID)	Session	9, 40	0.97	0.48	0.18	[0.62, 1.00]
E	Trials to criterion	Session	9, 40	6.50	1.2×10^{-5}	0.59	[0.67, 0.91]
F	Go probability	Session	9, 80	10.91	6.8×10^{-11}	0.55	[0.84, 0.94]
		Trial configuration	1, 80	514.67	1.4×10^{-36}	0.87	[0.99, 1.00]
		Session × Trial configuration	9, 80	10.90	7.1×10^{-11}	0.55	[0.84, 0.94]
G	Accuracy (Non-match)	Session	9, 67	5.10	3.0×10^{-5}	0.41	[0.60, 0.89]
		Group	1, 67	76.44	1.1×10^{-12}	0.53	[0.97, 0.99]
		Session × Group	9, 67	2.83	0.007	0.28	[0.19, 0.82]
	Accuracy (Cue-ID)	Session	9, 67	15.16	4.2×10^{-13}	0.67	[0.89, 0.96]
		Group	1, 67	3.55	0.064	0.05	[0.94, 1.00]
		Session × Group	9, 67	0.64	0.75	0.08	[0.48, 1.00]
H	Trials to criterion	Session	9, 67	34.18	1.1×10^{-21}	0.82	[0.95, 0.98]
		Group	1, 67	2.50	0.12	0.04	[0.93, 1.00]
		Session × Group	9, 67	7.78	9.7×10^{-8}	0.51	[0.76, 0.92]
I	Go probability (Exp)	Session	9, 78	8.28	1.5×10^{-8}	0.49	[0.78, 0.93]
		Trial configuration	1, 78	103.15	6.3×10^{-16}	0.57	[0.98, 0.99]
		Session × Condition	9, 78	4.41	1.1×10^{-4}	0.34	[0.53, 0.88]
	Go probability (Control)	Session	9, 56	9.77	8.3×10^{-9}	0.61	[0.81, 0.94]
		Condition	1, 56	0.03	0.86	0.001	[0.00, 0.78]
		Session × Trial configuration	9, 56	0.09	1.00	0.02	[0.00, 1.00]

Supplementary Table 1. *ANOVA results for Fig.2.*

Experimental group		Rule switching					Control			
Problem	Rat / Session	1	2	3	4	5	1	2	3	4
Non-match A	1th	45	44	39	40	26				
	2nd	55	42	34	40	19				
	3rd	44	41	38	45	28				
	4th	48	37	38	48	27				
	Last	49	46	55	29	27				
Non-match B	1th	42	54	50	33	22				
	2nd	41	47	57	35	25				
	3rd	31	48	50	47	26				
	4th	42	50	54	46	26				
	Last	45	38	58	43	22				
Non-match A2	1th	41	41	69	53	24				
	2nd	65	45	42	40	21				
	3rd	49	52	39	43	24				
	4th	53	42	41	46	28				
	Last	52	40	54	57	24				
Cue-id A	1th	49	38	51	37	30	29	22	28	22
	2nd	43	45	52	42	30	32	37	36	40
	3rd	48	49	60	47	28	29	24	47	43
	4th	40	56	62	30	27	32	26	31	34
	Last	56	48	57	36	25	26	21	21	37
Cue-id B	1th	43	51	50	37	32	23	31	17	26
	2nd	53	54	43	35	36		23	26	38
	3rd	53	47	48	41	32	28	17	21	56
	4th	48	52	42		30		17	21	30
	Last	54	47	47	46	27	28	12	23	53

Supplementary Table 2. *Number of recorded units for each rat and session.*

Problem	Session	t(df)	p	Cohen's d	95% CI
Non-match A	1st	t(193) = 1.04	0.30	0.07	[–0.01, 0.04]
	Last	t(205) = 2.56	0.011	0.18	[0.02, 0.13]
Non-match B	1st	t(200) = 2.88	0.004	0.20	[0.02, 0.12]
	Last	t(205) = 3.58	< 0.001	0.25	[0.06, 0.20]

Supplementary Table 3. *paired t-tests, comparing the mean firing rate of individual neurons for fig.3b*

Panel	Comparison	KS statistic (D)	p-value	n_1	n_2
Cue-id A 1st	**Non-match rule**	**0.260**	**0.0022**	**159**	**92**
	Cue-id rule	0.127	1.0	159	92
Cue-id A last	**Non-match rule**	**0.214**	**0.0255**	**166**	**96**
	Cue-id rule	0.183	0.1152	166	96
Cue-id B 1st	**Non-match rule**	**0.226**	**0.0223**	**167**	**83**
	Cue-id rule	0.160	0.3954	167	83
Cue-id B last	**Non-match rule**	**0.216**	**0.0267**	**159**	**93**
	**Cue-id rule**	**0.210**	**0.0377**	**159**	**93**

Supplementary Table 4. *Kolmogorov–Smirnov tests results comparing distributions of delta z-scores for fig.4*

Measure	Effect	df	F	p-value	partial η^2	95% CI
Proportion of Non-match rule selective neurons	Session	9, 67	1.90	0.067	0.203	[0.762, 1.0]
	**Group**	**1, 67**	**14.46**	**0.0003**	**0.177**	**[0.749, 0.972]**
	Session × Group	9, 67	0.51	0.865	0.064	[0.379, 1.0]
Proportion of cue-id rule selective neurons	Session	9, 67	1.78	0.088	0.193	[0.751, 1.0]
	Group	1, 67	0.04	0.841	0.001	[0.789, 1.0]
	Session × Group	9, 67	0.13	0.999	0.017	[0.000, 1.0]
Proportion of both rules' selective neurons	Session	9, 67	0.69	0.717	0.085	[0.506, 1.0]
	**Group**	**1, 67**	**6.99**	**0.010**	**0.094**	**[0.272, 0.956]**
	Session × Group	9, 67	0.46	0.899	0.058	[0.325, 1.0]

Supplementary Table 5. *ANOVA results for fig.4.*

Problem	Session	Non-match distance	Cue-id distance
Non-match A	1st	0.4602	0.088
	2nd	**0.0048**	1.000
	3rd	**<0.001**	0.108
	4th	**<0.001**	1.000
	Last	**<0.001**	1.000
Non-match B	1st	**<0.001**	1.000
	2nd	**<0.001**	1.000
	3rd	**<0.001**	1.000
	4th	**<0.001**	1.000
	Last	**<0.001**	1.000
Cue-id A	1st	**<0.001**	**<0.001**
	2nd	**<0.001**	**<0.001**
	3rd	**<0.001**	**<0.001**
	4th	**0.0092**	**<0.001**
	Last	**<0.001**	**<0.001**
Cue-id B	1st	**<0.001**	**<0.001**
	2nd	**<0.001**	**<0.001**
	3rd	**<0.001**	**<0.001**
	4th	**0.0220**	**<0.001**
	Last	**<0.001**	**<0.001**
Cue-id A Control	1st	1.0000	1.000
	2nd	0.4602	**<0.001**
	3rd	0.0880	**<0.001**
	4th	0.3188	**<0.001**
	Last	0.4550	**<0.001**
Cue-id B Control	1st	**0.0472**	**<0.001**
	2nd	0.1547	**<0.001**
	3rd	0.1275	**<0.001**
	4th	0.8947	**<0.001**
	Last	**<0.001**	**<0.001**

Supplementary Table 6. *Permutation test results for fig. 5d-e.*

Problem	Session	Non-match decoder	Cue-id decoder
Cue-id A	1st	**<0.001**	1.000
	2nd	**<0.001**	**<0.001**
	3rd	**0.0066**	1.000
	4th	0.8624	0.4068
	Last	**0.0100**	**<0.001**
Cue-id B	1st	**<0.001**	**<0.001**
	2nd	**<0.001**	**<0.001**
	3rd	1.000	1.000
	4th	0.8624	**<0.001**
	Last	1.000	1.000

Supplementary Table 7. *Significance level for comparing distances of UMAP centroids between experimental groups vs differences between null distributions.*

Panel	Measure	Effect	df	F	p-value	Partial η^2
F	CCGP (Non-match)	Session	9,40	122.423	3.061E-26	0.965
	CCGP (cue-identity)	Session	9,40	4.362	0.0005	0.495
G	CCGP (Non-match) Experiment	Session	9,39	5.05	1.56E-04	0.381
	CCGP (cue-identity) Experiment	Session	9,39	127.13	4.64E-26	0.97
	CCGP (Non-match) Control	Session	9,28	1.918	0.091	0.381
	CCGP (cue-identity) Control	Session	9,28	26.34	2.21E-11	0.894
H	Axis cosine (NM↔CI)	Session	9,40	5.323	8.869E-05	0.545
I	Axis cosine (NM↔CI) Experiment	Session	9,39	29.85	8.46E-15	0.87
	Axis cosine (NM↔CI) Control	Session	9,28	3.36	0.007	0.519

Supplementary Table 8. *ANOVA results for fig. 5f-i*

Measure	Effect	df	F	p-value	partial η^2	95% CI
Similarity to 'Non-match template'	Session	4, 35	1.08	0.381	0.110	[0.757, 1.0]
	**Group**	**1, 35**	**14.28**	**0.0006**	**0.290**	**[0.972, 0.721]**
	Session × Group	4, 35	0.99	0.424	0.102	[0.745, 1.0]
Similarity to 'cue-id template'	**Session**	**4, 35**	**5.12**	**0.0024**	**0.369**	**[0.913, 0.420]**
	Group	1, 35	0.06	0.811	0.002	[0.805, 1.0]
	Session × Group	4, 35	0.97	0.435	0.100	[0.742, 1.0]
Similarity to 'both rules template'	Session	4, 35	0.59	0.669	0.064	[0.660, 1.0]
	**Group**	**1, 35**	**5.61**	**0.024**	**0.138**	**[0.951, 0.022]**
	Session × Group	4, 35	0.10	0.981	0.012	[0.000, 1.0]

Supplementary Table 9. *ANOVA results for fig.6*

Group	Problem	Rule	Test type	Session effects	R^2	β	t	p	partial η^2	95% CI (η^2)
Experimental	Non-match A	Non-match	General	–	0.572	0.97	5.55	<0.001	0.57	[0.35, 0.73]
			With sessions	Session p=0.39; Interaction p=0.84	0.747	0.29	0.95	0.35	0.04	[0.00, 0.20]
		Cue-id	General	–	0.123	−0.13	−1.79	0.086	0.12	[0.00, 0.31]
			With sessions	Session p=0.62; Interaction p=0.51	0.231	−0.09	−0.72	0.48	0.02	[0.00, 0.13]
	Non-match B	Non-match	General	–	0.495	0.75	4.75	<0.001	0.50	[0.25, 0.70]
			With sessions	Session p=0.85; Interaction p=0.81	0.510	1.01	1.04	0.31	0.05	[0.00, 0.22]
		Cue-id	General	–	0.001	0.02	0.12	0.90	0.00	[0.00, 0.02]
			With sessions	Session p=0.71; Interaction p=0.72	0.009	−0.25	−0.35	0.73	0.01	[0.00, 0.09]
	Cue-id A	Non-match	General	–	0.226	0.35	2.59	0.016	0.23	[0.04, 0.45]
			With sessions	Session p=0.005; Interaction p=0.001	0.708	4.32	4.01	0.001	0.43	[0.19, 0.63]
		Cue-id	General	–	0.887	0.92	13.4	<0.001	0.89	[0.77, 0.95]
			With sessions	Session p=0.042; Interaction p=0.18	0.945	1.61	2.56	0.018	0.24	[0.04, 0.46]
	Cue-id B	Non-match	General	–	0.099	−0.12	−1.55	0.14	0.10	[0.00, 0.28]
			With sessions	Session p=0.75; Interaction p=0.92	0.356	0.00	0.002	0.999	0.00	[0.00, 0.00]
		Cue-id	General	–	0.557	0.61	5.26	<0.001	0.55	[0.31, 0.72]
			With sessions	Session p=0.020; Interaction p=0.045	0.767	3.40	2.51	0.021	0.23	[0.03, 0.45]
Control	Cue-id A	Non-match	General	–	0.002	−0.07	−0.21	0.84	0.00	[0.00, 0.02]
			With sessions	Session p=0.24; Interaction p=0.25	0.097	−11.1	−1.21	0.25	0.08	[0.00, 0.26]
		Cue-id	General	–	0.131	0.47	1.65	0.12	0.13	[0.00, 0.33]
			With sessions	Session p=0.015; Interaction p=0.068	0.707	7.67	1.98	0.065	0.20	[0.00, 0.41]
	Cue-id B	Non-match	General	–	0.102	0.17	1.35	0.20	0.10	[0.00, 0.29]
			With sessions	Session p=0.10; Interaction p=0.12	0.337	5.92	1.70	0.11	0.17	[0.00, 0.37]
		Cue-id	General	–	0.255	0.62	2.34	0.033	0.25	[0.02, 0.47]
			With sessions	Session p=0.001; Interaction p=0.003	0.725	15.8	3.71	0.002	0.50	[0.20, 0.70]

Supplementary Table 10. *Regression results for fig.7a-c*

Session	Slope	Correlation (r)
1st	0.89	0.86
2nd	0.37	0.95
3rd	0.03	0.06
4th	–0.46	–0.58
Last	–0.34	–0.87
**Early mean (1st-2nd)**	**0.63**	
**Late mean (3rd- last)**	**–0.26**	
**Fisher's z (early vs. late)**		$z = 4.20$, $p = 2.7 \times 10^{-5}$

Supplementary Table 11. *Slope analysis for fig. 7b*

Dependent variable	R^2	F (df=1,32)	p (slope)	β (slope)	95% CI for β	p (β)
Probability to ignore 'go' prediction	0.27	11.98	0.002	1.14	[0.47, 1.82]	0.002
Accuracy by cue-id rule	0.34	16.67	P<0.001	0.40	[0.20, 0.60]	<0.001

Supplementary Table 12. *Regression results for fig.7d,e*

Session	r	p
1st	−0.79	0.114
2nd	−0.75	0.142
3rd	−0.24	0.699
4th	0.06	0.923
Last	−0.29	0.637

Supplementary Table 13. *Pearson correlations for fig.7f*

Panel	Measure	Effect	df	F	p-value	Partial η^2	95% CI for η^2
E	Accuracy (non-match)	**Session**	**4,40**	**3.79**	**0.011**	**0.275**	**[0.185, 0.890]**
		Group	1,40	1.30	0.261	0.031	[0.906, 1.000]
		Session × Group	4,40	0.15	0.960	0.015	[0.205, 1.000]
	Accuracy (cue-ID)	Session	4,40	0.99	0.423	0.090	[0.744, 1.000]
		Group	1,40	0.77	0.385	0.019	[0.890, 1.000]
		Session × Group	4,40	0.58	0.678	0.055	[0.655, 1.000]
F	Accuracy (non-match)	Session	4,40	1.40	0.252	0.123	[0.791, 1.000]
		**Group**	**1,40**	**31.16**	**2×10^{-6}**	**0.438**	**[0.913, 0.984]**
		Session × Group	4,40	0.02	0.999	0.002	[0.000, 1.000]
	Accuracy (cue-ID)	Session	4,40	0.31	0.868	0.030	[0.510, 1.000]
		Group	1,40	0.53	0.469	0.013	[0.878, 1.000]
		Session × Group	4,40	1.24	0.310	0.110	[0.775, 1.000]
G	Accuracy (non-match)	**Session**	**4,40**	**4.20**	**0.006**	**0.296**	**[0.277, 0.898]**
		**Group**	**1,40**	**12.07**	**0.001**	**0.232**	**[0.647, 0.969]**
		**Session × Group**	**4,40**	**7.28**	**0.0002**	**0.421**	**[0.640, 0.933]**
	Accuracy (cue-ID)	**Session**	**4,40**	**67.81**	**2.8×10^{-17}**	**0.871**	**[0.974, 0.991]**
		**Group**	**1,40**	**111.7**	**3.8×10^{-13}**	**0.736**	**[0.983, 0.995]**
		**Session × Group**	**4,40**	**5.46**	**0.001**	**0.353**	**[0.478, 0.916]**
H	Accuracy (non-match)	**Session**	**4,40**	**3.47**	**0.016**	**0.258**	**[0.103, 0.883]**
		**Group**	**1,40**	**20.68**	**4.9×10^{-5}**	**0.341**	**[0.844, 0.978]**
		Session × Group	4,40	0.98	0.431	0.089	[0.742, 1.000]
	Accuracy (cue-ID)	**Session**	4,40	**7.72**	**0.0001**	**0.436**	**[0.666, 0.936]**
		**Group**	**1,40**	**31.33**	**2×10^{-6}**	**0.439**	**[0.914, 0.984]**
		Session × Group	4,40	0.15	0.960	0.015	[0.206, 1.000]

Supplementary Table 14. *ANOVA results for fig.8.*

Problem	Session	Non-match decoder	Cue-id decoder
Non-match A 1	1th	0.037031	0.011675
	2nd	**<0.001**	0.001541
	3rd	**<0.001**	0.404709
	4th	**<0.001**	0.024948
	Last	**<0.001**	0.085844
Non-match B	1th	**<0.001**	0.001919
	2nd	**<0.001**	0.675558
	3rd	**<0.001**	0.591149
	4th	**<0.001**	0.244685
	Last	**<0.001**	1.000000
Cue-id A	1th	**<0.001**	**<0.001**
	2nd	**<0.001**	**<0.001**
	3rd	**<0.001**	**<0.001**
	4th	0.021711	**<0.001**
	Last	**<0.001**	**<0.001**
Cue-id B	1th	0.007446	**<0.001**
	2nd	**<0.001**	**<0.001**
	3rd	**<0.001**	**<0.001**
	4th	**<0.001**	**<0.001**
	Last	**<0.001**	**<0.001**
Cue-id A Control	1th	1.000000	**<0.001**
	2nd	0.262806	**<0.001**
	3rd	0.026859	**<0.001**
	4th	0.067862	**<0.001**
	Last	0.002775	**<0.001**
Cue-id B Control	1th	0.382626	**<0.001**
	2nd	0.006006	**<0.001**
	3rd	0.284917	**<0.001**
	4th	0.004122	**<0.001**
	Last	0.026580	**<0.001**

Supplementary Table 15. *Combined p values for Supplementary figure 6.*

Effect	df	F	p-value	Sig.
Group	1, 240	84.639	$1.80×10^{-17}$	***
Sex	1, 240	3.775	0.0532	n.s.
Session	19, 240	42.737	$5.96×10^{-66}$	***
Rule	1, 240	28.001	$2.73×10^{-7}$	***
Group × Sex	1, 240	1.846	0.1755	n.s.
Group × Session	19, 240	2.796	$1.47×10^{-4}$	***
Sex × Session	19, 240	0.845	0.6520	n.s.
Group × Rule	1, 240	11.392	$8.60×10^{-4}$	***
Sex × Rule	1, 240	2.077	0.1509	n.s.
Session × Rule	19, 240	78.924	$9.30×10^{-92}$	***
Group × Sex × Session	19, 240	0.562	0.9302	n.s.
Group × Sex × Rule	1, 240	0.487	0.4858	n.s.
Group × Session × Rule	19, 240	6.229	$6.64×10^{-13}$	***
Sex × Session × Rule	19, 240	1.951	0.0116	*
Group × Sex × Session × Rule	19, 240	0.749	0.7658	n.s.
Post-hoc Sex interactions (per session × rule)	-	-	All Bonferroni-adjusted p > 0.05	n.s

Supplementary Table 16. *ANOVA results for sex difference in the inactivation experiment.*
